# Telemedicine in Pediatric Infectious Diseases

**DOI:** 10.3390/children8040260

**Published:** 2021-03-28

**Authors:** Marco Pappalardo, Umberto Fanelli, Vincenzo Chiné, Cosimo Neglia, Andrea Gramegna, Alberto Argentiero, Susanna Esposito

**Affiliations:** 1 Pediatric Clinic, Pietro Barilla Children’s Hospital, Department of Medicine and Surgery, University of Parma, 43126 Parma, Italy; marco.pappalardo@studenti.unipr.it (M.P.); umbertaker@msn.com (U.F.); chinevincenzo@gmail.com (V.C.); negliamino@gmail.com (C.N.); aargentiero85@gmail.com (A.A.); 2 Respiratory Unit and Cystic Fibrosis Adult Center, Internal Medicine Department, Fondazione IRCCS Ca’ Granda Ospedale Maggiore Policlinico, 20122 Milan, Italy; andrea.gramegna@unimi.its; 3 Department of Pathophysiology and Transplantation, University of Milan, 20122 Milan, Italy

**Keywords:** antimicrobial stewardship, COVID-19, pediatric infectious disease, telemedicine

## Abstract

Telemedicine is the remote practice of medicine through the use of information and communication technologies for the prevention, diagnosis, treatment and management of diseases. In this narrative review, we illustrate how telemedicine technologies are increasingly integrated into pediatric infectious disease programs with the aim of facilitating access to specialist care and reducing costs. There is widespread use of telemedicine for the management of acute and chronic infectious diseases, particularly in countries in which the majority of the population lives in rural areas, far from third-level hospital centers located in large urban centers. Obviously, telemedicine is also used in developed countries, and its importance has been further increased recently given the COVID-19 pandemic. It has many advantages for patients, such as saving time, money and working hours, and reducing cancelled appointments and delays, while there are also many advantages for doctors, allowing collaborations with specialists and continuous updating. Among the disadvantages are the limitation in carrying out an objective examination, which is particularly important for children under 2 years of age, and the need for cutting-edge technology and reliable connectivity. Telemedicine increasingly represents the future and the beginning of a new healthcare system that also will redefine medical care for the treatment of infectious diseases, both acute and chronic. However, the majority of the experience has involved adults, and its validation in pediatric care, as well as its application in real-life practices, are urgently needed.

## 1. Introduction

Telemedicine, also known as telehealth, is the remote practice of medicine through the use of information and communication technologies for the prevention, diagnosis, treatment and management of diseases [1]. Communications can be synchronous (e.g., a video call in which the two parties communicate in real time) or asynchronous (e.g., email) [2]. The wide availability of broadband Internet has gradually expanded the potential of telemedicine, with the possibility of facilitating interactions between patients and doctors and between two or more doctors. Telemedicine facilitates patients’ access to specialist doctors (e.g., e-visits, e-consultations), particularly in areas far away from large hospital centers, where alternative methods need to be developed to provide adequate health services [2,3].

Telemedicine can also be used to provide continuing medical education and access to programs and resources for small or rural hospitals (e.g., antimicrobial remote assistance) [4]. For these reasons, many studies cite common arguments to support the implementation of telemedicine programs: telemedicine promises greater access to treatment, more widespread treatment and excellent cost-effectiveness [4,5,6,7].

The first applications of telemedicine can be traced back to the beginning of the 1900s: in 1929, it was possible to send X-rays by telegram [8]. Starting in the 1970s, it was also possible to transmit radiographic images over 20 miles away by microwaves [9]. The first example of the application of telemedicine in clinical practice dates back to 1975, when a working group from a Massachusetts school compared classic telephone communication between hospital doctors and nurses in remote areas with the then-innovative television consultation. The study showed that the latter lasted longer (50 min versus 40 min by telephone) but allowed the doctor to postpone a face-to-face evaluation while achieving a similar rate of patient satisfaction [10]. Following these first evaluations, the rapid evolution of information and communication technology awakened interest in telemedicine as a reliable and usable solution in the diagnostic–therapeutic field [11,12,13,14,15,16,17].

Today, as technology advances, the potential of telemedicine has expanded in innovative directions. Approaches incorporate new technologies, such as high-definition cameras, encryption software and electronic stethoscopes, otoscopes and ophthalmoscopes to treat a large number of both acute and chronic infections [4,5,6,7]. In this narrative review, we will illustrate how telemedicine technologies are increasingly integrated into pediatric infectious-disease programs with the aim of facilitating access to specialist care and reducing costs [18]. We reviewed the literature by searching the Medline database via the PubMed interface and Google Scholar for articles published between January 2010 and December 2020. The keywords used were “telemedicine” AND “infection” OR “infectious disease” OR “antibiotic” OR “antimicrobial” OR “COVID” OR “SARS-CoV-2” AND “children” OR “paediatric” OR “pediatric”.

## 2. Management of Acute Infections

Telemedicine technologies have helped us diagnose, treat and follow patients at a distance who are suffering from acute infectious diseases such as community-acquired pneumonia, upper respiratory tract infections, skin and soft tissue infections, urinary tract infections and bacterial endocarditis [19]. One of the advantages of following patients with acute infections by telemedicine is to reduce the durations of hospitalization and antibiotic therapy, as demonstrated by a 2008 US retrospective study conducted on three infectious diseases: community-acquired pneumonia, febrile neutropenia and skin-wound infections [20].

Telemedicine appears to be essential in countries in which the majority of the population lives in rural areas, far from third-level hospital centers located in large urban centers. A four-year retrospective study conducted in India in 2009 on 431 pediatric patients showed a gradual increase in the number of telemedicine consultations with specialists, based on clinical news, laboratory surveys and radiological images, with a high rate of satisfaction on the part of parents due to the significant savings in money and time (e.g., travel expenses for the patient and the accompanying person, accommodation near the hospital and daily expenses for each consultation) [3]. Similarly, in sub-Saharan Africa, high infant-mortality rates are observed, mainly due to acute respiratory infections, diarrheal diseases and malaria [21]. In these areas, access to services is still limited, and the long distance to healthcare facilities contributes negatively to children’s health [22]. A study conducted in Ghana in 2018 assessed how the use of mobile devices (known as mobile Health, mHealth) in support of public-health measures could improve communication between patients and medical specialists [23]. The results of studies in Zambia and Pakistan reduced mortality in childhood pneumonia due to calling “Integrated Community Case Management”, based on community health workers [24,25]. MHealth, via easy-to-use software on mobile phones, could help community health workers to identify children with pneumonia who need appropriate therapy, and children who need closer monitoring. In addition, mHealth automated tools could take into account several predictors, such as young age, age-specific respiratory rate, oxygen saturation, malnutrition and comorbidities, to predict the failure of oral antibiotic therapy in children with pneumonia [26]. Furthermore, when adding a mobile healthcare application with a pediatric breath meter and pulse oximeter, it is possible to improve adherence to diagnostic protocols, incorporate physiological measurements such as respiratory rate and pulse oximetry, and provide general decision support [27].

Telemedicine can also play a role in disease surveillance in rural areas in which delays in disease recognition and intervention can lead to an uncontrolled spread of infectious disease. In 2018, in northern Uganda, real-time health surveillance of an epidemic of Nodding syndrome, an epileptic disorder likely to be the result of infestation with the nematode *Onchocerca volvulus*, was conducted [28]. Using smartphones and a personalized data-collection platform, a real-time electronic map of children’s health in rural northern Uganda was developed for a period of three consecutive months using data from 10 villages strongly affected by Nodding syndrome. The surveillance system was based on weekly reports of neurological events via smartphones managed by mHealth reporters living in the communities under investigation. Viewed on a map, the collected data provided real-time health geography that helped ensure drug availability and the rapid reporting of new cases in a region in which that was previously impossible.

Obviously, telemedicine is also used in developed countries. A recent study carried out in the US in 2019 compared the quality of the prescription of antibiotics for acute respiratory infections in children in three different contexts: direct consumer telemedicine, first aid and general practice. The authors reported a higher number of prescriptions for antibiotics with less adherence to the guidelines for telemedicine visits than the other modalities. Despite these unsatisfactory results, telemedicine is recognized as having wide margins for improvement and some mitigating factors. In particular, the information transmitted in telemedicine was limited because the personal devices used by parents did not incorporate peripheral attachments designed to improve remote visits (e.g., teleotoscopy) [29]. Remote monitoring of physical activity, temperature and other parameters permit the acquisition of information in a home setting. Telemedicine can also offer considerable advantages in the hospital environment. To ensure the faster and more careful management of sepsis, Machado et al. implemented a sepsis alert system in the emergency room of a US hospital. The alarm activated a team of doctors and a cart that was called an “intensive care unit on wheels”, which allowed a direct connection between the patient and an intensivist, who supervised the management and treatment of sepsis elsewhere in the hospital. In adults, this technological innovation has led to faster antibiotic delivery times and reduced hospitalization duration, with positive effects on mortality and healthcare costs [30].

In conclusion, telemedicine is useful in different settings for management of acute infectious diseases in children. However, its use has the major limitation that the patient must be in a stable clinical condition. Patients in critical condition require frequent monitoring and urgent medical interventions, so they are not good candidates for telemedicine [5].

## 3. Management of Chronic Infections

The use of telemedicine for the treatment of chronic diseases has been a growing sector in recent decades, and such use has been described in the management of cardiac, pulmonary, renal and many other organ pathologies [31]. Telemedicine is also used to manage patients with chronic infectious diseases such as HIV, HCV, and tuberculosis [32]. Assuming the patient is stable, with this method, it is possible to carry out a specialist follow-up for infectious diseases at regular intervals, such as every 6–12 months, with improved therapeutic adherence and clinical laboratory benefits [5]. However, the “optimal” frequency of consultations has never been studied in depth. The creation of complex algorithms using patient questions/answers, combined with the progress made in the field of artificial intelligence, should lead to an intervention by specialists only in truly urgent situations [1].

A very important development in African countries was the use of e-health prevention of mother-to-child transmission (PMTCT) interventions against HIV, useful for improving maternal retention, defined as the establishment and maintenance of a stable relationship between the person with HIV and a care team, adherence to antiretroviral treatment and reduction of the risk of HIV infection in neonates [33,34]. A systematic review on PMTCT interventions for pre- and postpartum retention considered 10 studies, all of which were from sub-Saharan Africa except one from the UK, and concluded that telephone interventions (SMS or calls) can improve retention, especially in the first three months after childbirth [35]. One of these studies was based on sending 14 SMS (eight prenatal, six postnatal) and found increased retention at eight weeks after childbirth [36]. A recent study carried out in Kenya assessed the effectiveness of prenatal telemedicine counseling among pregnant women with HIV [37]. Between May 2013 and September 2015, 404 HIV-positive pregnant women were recruited and assigned randomly to the intervention or control group. The intervention included a fixed protocol of telephone calls by a counselor to provide individual support as needed. Personalized telephone counseling has proven to be very effective in keeping HIV-positive mothers in care, in promoting HIV testing for infants, who were less likely to test positive, and in performing postnatal treatment [37]. The mHealth interventions certainly have advantages with regard to promoting adherence to treatment for women during and beyond pregnancy and the post-partum period. The SMSs follow a program designed to encourage adherence to antiretroviral therapy while recognizing specific concerns about key events in pregnancy and the postpartum period, such as neonatal prophylaxis and weaning [38]. The mHealth interventions for PMTCT can be particularly effective when initiated during pregnancy, motivating women to make behavioral changes that benefit their health and that of their child [39]. However, there are several problems that e-health systems face with regard to PMTCT. Because the date of birth is not perfectly predictable, it is difficult to precisely time messages related to birth planning, childbirth and the immediate postpartum period. Moreover, given the poverty of these populations, access to mobile phones is often limited, and there are considerable difficulties in contacting mothers, as well as poor adherence to antiretroviral therapy in a significant percentage of the population.

Another example of the use of telemedicine to improve HIV management is based on the Extension for Community Healthcare Outcomes (ECHO) model, in which nonmedical staff are trained via distance learning with targeted lectures and the discussion of clinical cases by university specialists. The ECHO project was recently implemented for the perinatal treatment of HIV, and has proven to be a valuable tool to effectively educate community providers caring for HIV-positive pregnant women [40]. In addition, telemedicine promises to extend access to specialist HIV treatment to isolated populations. For example, in the UK and Ireland, a team of multidisciplinary specialists asynchronously reviewed the cases of a regional network of general pediatricians treating infants and children perinatally infected with HIV on a monthly basis, enabling them to treat children in environments close to their homes, which were often rural and far from urban centers [41].

A recent and future field of application in telemedicine aims to combat cytomegalovirus (CMV) retinitis, a formidable complication of AIDS and the leading cause of blindness in HIV-infected populations with limited access to treatment. Taking advantage of teleophthalmology studies on the diagnosis of diabetic retinopathy and retinopathy of prematurity, two studies in Thailand used eyeball photography with asynchronous evaluation from remote raters for CMV screening; however, the results were not fully satisfactory [42,43].

Infectious diseases can also complicate chronic pathologies present in the pediatric population, even if they are present in lower percentages than in adults. An example is cystic fibrosis (CF). Lung disease in CF is characterized by dense secretions colonized by bacteria, resulting in inflammation and chronic infection [44,45]. Recurrent exacerbations of lung infections in CF patients cause progressive lung damage, and these exacerbations impose economic and social pressures on patients and their families. Antimicrobial parenteral therapy and hospitalization are considered the gold standard of pulmonary-exacerbation management. A study conducted in a large US center on children with CF tried to apply telemedicine in the management of oral antibiotic therapy in pediatric patients with mild to moderate relapses. A modified form of the Akron Children’s Hospital CF Pulmonary Exacerbation Score (PES) has been adopted as a telephone triage tool [46]. The telephone PES used systemic signs, including fever, fatigue, appetite, absence from school and increased lung work; signs such as coughing, dyspnea and hemoptysis, although without objective data (e.g., results of lung-function tests, weight); and objective examination results. The reported symptoms were assigned an individual weighted score from 0 to 16, and a multidisciplinary pediatric CF care team (which included a nurse, pharmacist, social worker, dietician, pulmonologist and respiratory physiotherapist) provided guidance for home/ambulatory or possibly hospital management. The authors were able to develop a close outpatient monitoring to prevent an irreversible decline in lung function [47].

Overall, considering the available evidence, telemedicine appears useful in different settings for management of chronic infections in children, with HIV and respiratory exacerbations in CF as the best examples. Further studies are needed on the optimal monitoring and follow-up.

## 4. Telemedicine and Antimicrobial Stewardship

Antimicrobial stewardship (AS) is a key element in improving the quality of hospital care. AS requires a multidisciplinary antimicrobial management team, including a prescribing physician, an infectious-disease specialist, a microbiologist and a pharmacist, to encourage accountability, drug competence, and the education of suppliers and patients [48,49].

Given their limited resources, some small or rural hospitals have chosen to compete with other third-level AS teams through telemedicine, commonly in the form of asynchronous communications, with varying degrees of success [6]. This approach is also needed in developing countries. A pediatric study was conducted in Bogota, Colombia, where most patients are concentrated in rural areas, often far from the main facilities [46]. The system has reduced the number of patient transfers to higher-level hospitals by 83%, as well as waiting times for transfers, improving healthcare at a reasonable cost. At the same time, an approximately 17% decrease in the use of antibiotics, which is an important current public-health issue, has been observed due to consultation with infectious-disease specialists [50].

Moreover, a remote telemedicine consultancy program has been developed in Italy between a pediatric cardiac hospital and a highly specialized infectious-disease center [18]. In addition to the biweekly discussion of all clinical cases, an antibiotic-therapy consultation was available for each patient via telemedicine. A comparison before and after the study showed a trend toward a reduction in the incidence rate of nosocomial infectious diseases, a reduction in the overall cost of antibiotics and a reduction in the selection for multidrug resistant bacteria, thus improving patient safety.

Available evidence suggests that telemedicine can allow AS to become a daily practice in different pediatric settings by improving the quality of care and preventing the emergence of resistance.

## 5. Telemedicine and Infection Prevention

Various strategies have been evaluated for the prevention or early detection of infectious diseases; for example, sending anonymous text messages to sexual partners in the case of HIV-positive test results or sending rapid tests automatically in the case of dating sites [1].

In tuberculosis, a screening tool based on a mobile phone has been created to measure the induration of the tuberculin skin test, which is used to detect latent tuberculosis in adults and active tuberculosis in children [51]. The tool used a mobile application developed on the Android platform to capture images of an induration, with subsequent reconstruction and 3D measurement. The results were remarkable and yielded more accurate results than the current pen-and-ruler method. The mobile application provided an alternative method of measurement that could eliminate the need for a personal check-up and improve the screening capacity for tubercular infection.

Moreover, another important application of telemedicine is in the field of immunization. Text messaging could be a new and easily applicable approach to promoting vaccines. A US study involved sending out reminders to promote influenza vaccination among children and adolescents. Simply sending a message was associated with a higher number of vaccines being administered than in previous years [52].

In conclusion, telemedicine also could have a role in infection prevention as a reminder for screening and vaccination, as well as a tool for the evaluation of test results.

## 6. Telemedicine and COVID-19

During the COVID-19 pandemic, telemedicine has emerged as a key technology to bring medical care to patients while reducing the transmission of severe acute respiratory syndrome coronavirus 2 (SARS-CoV-2) [53]. The response to viral spread included early diagnosis, patient isolation, symptomatic contact monitoring, monitoring of suspected and confirmed cases, and quarantine. In this context, telemedicine (in particular video consultations) was promoted and expanded to counter the spread of the virus, especially in the UK and the US [54]. In the US, between 2 March and 14 April 2020, telemedicine visits for urgent care increased by 135%, while nonurgent telemedicine visits increased by 4345% [55].

Telemedicine has proven useful in previous outbreaks of diseases caused by coronaviruses, such as SARS-CoV (the coronavirus associated with severe acute respiratory syndrome) and MERS-CoV (the coronavirus associated with Middle East respiratory syndrome) [56], and outbreaks due to the Ebola and Zika viruses [57]. During the COVID-19 pandemic, because of technological improvements and the widespread use of smartphones and applications (e.g., WhatsApp, Skype or Facetime), tele-expertise, home video monitoring of patients and teleconsultation for the triage of suspicious cases were performed. During a home video interaction, the patient can interact with a triage operator who collects an accurate anamnesis and performs an observational evaluation [58]. The evaluation should include observation of the general aspect (searching for diaphoresis, paleness or redness), calculation of the respiratory rate and work, evaluation of the oropharynx (looking for the presence of erythema, exudate or tonsillar hypertrophy) and a search for lymphadenopathy. On the basis of symptomatology and comorbidities, the patient can be directed to the appropriate testing facility if necessary.

In pediatrics, the already-complex doctor–patient communication is further complicated by telecommunication [59]. In our experience, we showed that during the COVID-19 outbreak, the use of telemedicine for the management of pediatric infectious diseases permitted patients to avoid hospital access in 90% of the cases, favoring reduction of the pressure on the hospitals [60]. However, Italy and many other countries do not have a regulatory framework that authorizes, integrates and reimburses telemedicine services, even in emergency and epidemic situations [61]. This is extremely important for ensuring continuity of care in patients with chronic diseases. Limitations that should be overcome include the lack of integration with the electronic medical records of the national health system, the lack of interconnection between telemedicine services operating at different levels, the lack of a real multidisciplinary approach to patient management, heavy privacy legislation, the lack of clear guidelines and the lack of reimbursements [62].

In conclusion, even if the presence of a pandemic is unfortunate, it must also be seen as an opportunity to increase telemedicine services, guaranteeing adequate and easily accessible healthcare for all patients, especially the most fragile. For countries that do not have telemedicine integrated into their national health system, the COVID-19 pandemic is an invitation to the widespread adoption of this practice [56].

## 7. Next Steps in Implementing Telemedicine in Real-World Practice

Telemedicine is a tool that provides numerous advantages in the management of pediatric infectious diseases. Table 1 summarizes the main advantages and disadvantages.

A primary advantage is greater accessibility to specialist care [1]. This is a key advantage for isolated populations, whose lack of access is one of the main obstacles to obtaining optimal care. Greater access to specialist care through telemedicine for patients with both acute and chronic infectious diseases allows many more patients to be promptly evaluated, triaged and treated. Experience in pediatrics is limited, whereas adult patients appeared satisfied with the level of privacy during appointments and the quality of the professional patient–healthcare provider relationship and feel more involved in their treatment [1]. Participation in remote appointments through telemedicine saves patients time, reduces the distance they have to travel and reduces the number of days of they have to be absent from work. Most patients indicated that they would choose to have their appointments via telemedicine again [6].

Another potential benefit is the cost-effectiveness of telemedicine. Studies have suggested that telemedicine is cost-effective, although further confirmation is needed [63]. Telemedicine is also advantageous for doctors, as its use is associated with reductions in canceled appointments and delays [1]. Telemedicine should not be seen as a simple teleconference, but as a tool with which experts from different specialties can evaluate a patient’s clinical and individual data in real time to solve complex problems [18].

Telemedicine has also been applied to inpatients with the objective of reducing the length of hospitalization, ensuring correct management of antibiotics, improving the quality of life of patients and maximizing the cost-effectiveness of patient management. The results seemed encouraging, but further studies are needed [64]. A similar approach could also be used to manage antibiotics at a territorial level, with AS interventions aimed at parental education and optimizing prescriptions. Francis et al. showed that the use of an interactive brochure on respiratory-tract infections in children could reduce consultations with a doctor and the use of antibiotics [65,66].

Inevitably, the main disadvantage of telemedicine compared to traditional visits is the impossibility of carrying out a complete objective physical examination. Objective examination during telemedicine consultations is particularly difficult, especially in children younger than 2 years of age or if the image quality of the telemedicine equipment is poor [1]. Moreover, the diagnosis of otitis media in children requires the visual inspection of the tympanic membrane [67], and an accurate diagnosis of streptococcal pharyngitis requires a rapid strep test [68]. Making these diagnoses via telemedicine in the absence of correct visualization may prove unreliable. In addition, other limits of telemedicine include the reliability of connectivity, need for high-speed or large bandwidth, legal and ethical issues, data security and patient confidentiality. All these issues can increase uncertainty among healthcare professionals and patients [69]. Furthermore, personal devices may vary in quality with regard to microphones, cameras and WiFi, and some visits are completed using only a phone. These problems reduce the clinical data in the doctor’s possession and can create difficulties during pediatric visits due to the limited ability of children to communicate symptoms [30]. Other obstacles to the development of telemedicine in pediatrics are the lack of a user-friendly interface to reassure the child, the technophobia of doctors, inadequate training in technology and concern about the quality of clinical care [3]. There are also some other issues: security and confidentiality, professional liability, licensing, reimbursement and medical–legal aspects of treatment recommendations. It is necessary to consider the medical–legal status of remote consultations, including the obligation for proper registration and documentation, the maintenance of the security of electronic files and the transmission of patient data [70].

In conclusion, as the COVID-19 pandemic has revealed, it is desirable that telemedicine should be used globally and integrated into the public-health response. These issues remain to be addressed and resolved: its integration into international and national guidelines with the definition of national regulations and funding frameworks; a standardized strategy to be applied in the event of any epidemic on a local, national or global scale; an operational plan to guide doctors to switch to outpatient teleconsultation and the remote monitoring of patients as needed; education of the population on the use of telemedicine; a sharing mechanism to be integrated with epidemiological surveillance; and accurate scientific evaluation and dedicated research funds to describe and assess the impact of telemedicine during epidemics.

## 8. Conclusions

Telemedicine increasingly represents the future and the beginning of a new healthcare system that also will redefine medical care for the treatment of infectious diseases, both acute and chronic. Its key benefit is increased access to treatment for isolated populations, high levels of satisfaction and cost savings. We expect a growing number of healthcare providers in the coming years will connect with patients remotely on a desktop or laptop computer. Doctors will select interactive videos and have continuous access to patient records, with patient information stored in archives accessible anywhere in the world. They will be able to consult online texts and diagnosis and treatment algorithms. This is why research into the safety, effectiveness, cost-effectiveness and patient and clinician satisfaction associated with telemedicine has become an absolute priority. However, the majority of the experience has involved adults, and its validation in pediatric care, as well as its application in real-life practices, are urgently needed.

## Figures and Tables

**Table 1:** Advantages and disadvantages of the use of telemedicine in pediatrics.

Advantages	Disadvantages
Access to specialist care for both acute and chronic infectious diseases	Inability to carry out an objective examination, which is especially important for children under 2 years of age
Visits for patients living in rural areas away from urban centers, saving time, money and work hours	Limited user-friendly interface with a child who is not always able to describe the symptoms; limited clinical data available to the doctor
Fewer cancelled appointments and delays	Need for cutting-edge technology, connectivity and reliability
For doctors, it is a tool that allows collaboration with specialists and continuous updating	Need for rules governing security and confidentiality, professional liability, licensing, reimbursement and medical–legal aspects of therapeutic recommendations
In the hospital environment, it is used for the correct management of antibiotics, and to improve patient quality of life and maximize cost-effectiveness	Lack of international and national guidelines to be applied in case of any epidemic on a local, national or global scale

## Data Availability

Not applicable for a review article.
